# Supplementary material for: Osteoblast-osteoclast co-cultures: A systematic review and map of available literature
Source: PLoS One. 2021 Nov 4;16(11):e0257724. doi: 10.1371/journal.pone.0257724 (PMC8568160; doi:10.1371/journal.pone.0257724)
Supplement: Attachment — Submitted filename: Response to Reviewers.docx [file pone.0257724.s007.docx]

# Reviewer comments

Reviewer #1: In this review Authors aim to create databases to facilitate research of methods concerning co-culture of OB and OC of all papers published up to 6 January 2020. The idea is very useful and the review is well organized. I any case, Authors should reduce it because it is too long and dispersive

Reviewer #2: The review is very interesting. The Authors made a tremendous work of research in scientific literature. It is well organized and can help researchers to choose the best protocol for their specific experimental demands. Although this, the Authors must make the manuscript more fluent. It is too long and, in such points, redundant.

The authors thank the reviewers for their positive approach to this manuscript and agree with their concerns regarding length and fluency. The manuscript has now been thoroughly shortened and reduced in length by more than 25%. This was achieved mainly by removing redundant or duplicate information and carefully rewriting each section of the manuscript in a concise manner while still describing the important content. We believe we have succeeded in improving readability and fluency, without significantly reducing the value that the manuscript provides.
